# An Explanation

**Published:** 1842-02

**Authors:** 


THE WESTERN JOURNAL.

Vol. V.—No. II.

LOUISVILLE, FEBRUARY 1, 1842.

### AN EXPLANATION.

We received Dr. Seaton's communication, in reply to the strictures on his essay on Milk-sickness, just as the last sheets of our January number were about going to the press, and sent it to the printers without reading it, in the full confidence that it did not contain any thing exceptionable. When we further state, that, by a misunderstanding, the proof-sheet was not submitted to the inspection of either of the senior editors, and that the junior editor did not feel at liberty to alter the language of the article, supposing it had received the approbation of the former, our readers will be able to comprehend how so extraordinary a paper found a place in the Journal. Our friend, Dr. S., we are sure, on reading his remarks in print, will be struck with the harshness, injustice, and impropriety of a number of expressions, which, assuredly, we should have felt constrained to suppress if we had read his communication. Our colleague, Professor D., our readers require not to be informed by us, is a writer of equal caution and experience, who in the statement of facts is as scrupulously exact, as he is acute and discriminating in observing them. Y.

